# Modeling the Thermal Inactivation of *Monascus ruber* Ascospores Isolated from Green Olive (*Arauco* Cultivar) Storage Brine: An Alternative Strategy to Reduce Antifungal Chemical Agents

**DOI:** 10.3390/foods13121881

**Published:** 2024-06-15

**Authors:** Leandro Pereira Cappato, Amanda Mattos Dias-Martins, Izadora Martina de Freitas Meireles, Elisa Helena da Rocha Ferreira, Wilson José Fernandes Lemos Junior, Amauri Rosenthal

**Affiliations:** 1 Instituto Federal Goiano, Rio Verde, Goiás 75901-970, Brazil; 2 Department of Food Technology, Federal Rural University of Rio de Janeiro, Seropedica 23897-970, Brazil; 3 Department of Biologia, University of Padova, 35020 Padua, Italy; 4 Embrapa Food Technology, Av. das Américas, Rio de Janeiro 23020-470, Brazil

**Keywords:** table olives, predictive microbiology, heat inactivation, heat-resistant molds

## Abstract

*Monascus ruber* is an important fungus that causes spoilage in table olives, resulting in the darkening of the brine, the softening of the fruit, increased pH, and apparent mycelial growth. This study aimed to evaluate this resistance, providing a model to determine the optimal processing conditions for mitigating fungal contamination and prolonging shelf life without antifungal agents while optimizing pasteurization to reduce energy consumption. The resistance in brine (3.5% NaCl; pH 3.5) from *Arauco* cultivar green olives imported from Argentina was assessed. Four predictive models (log linear, log linear + shoulder, log linear + tail, log linear + shoulder + tail) estimated kinetic parameters for each survival curve. Log linear + shoulder + tail provided the best fit for 70 °C and 75 °C, with low RMSE (0.171 and 0.112) and high R^2^ values (0.98 and 0.99), respectively, while the log linear model was used for 80 °C. Decimal reduction times at 70, 75, and 80 °C were 24.8, 5.4, and 1.6 min, respectively, with a z-value of 8.2 °C. The current regulatory processes are insufficient to eliminate *M. ruber* at requisite levels, considering reduced antifungal agents.

## 1. Introduction

To find sustainable solutions in table olive production, reducing preservatives can be considered to meet consumer demand for cleaner labels. However, this increases the risk of fungal contamination, leading to spoilage and the production of mycotoxins, which ultimately compromise the safety and quality of the products [1]. Besides that, producers must implement stringent quality control measures and optimize processing conditions as part of a sustainable solution. Exploring alternative preservation methods, such as optimizing thermal processing, can also be beneficial by reducing energy consumption and enhancing preservation effectiveness, thereby improving the quality and safety of commercialized olives. Balancing preservative reduction with effective fungal control ensures product safety and meets consumer demands for cleaner-label olive products [2].

Fungus can lead to physical changes in table olives, including discoloration, texture alterations, and off-flavors, rendering them unpalatable or unsuitable for consumption [3]. Additionally, some fungal species produce mycotoxins, which are toxic secondary metabolites that pose serious health risks to consumers if ingested. Moreover, the costs associated with implementing measures to prevent and control fungal contamination, such as improved sanitation practices, monitoring systems, and quality control measures, further contribute to the economic burden on the table olive industry [4]. To mitigate fungal contamination and minimize associated losses, olive producers and processors must implement comprehensive strategies for disease management and quality assurance [3].

Heat-resistant molds, particularly those within the *Ascomycetes* class, like *Byssochlamys fulva*, *B. nivea*, *Talaromyces flavus*, and *Neosartorya fischeri*, are notable for their ability to produce ascospores. These fungi are among the most important deteriorating microorganisms present in processed fruits and vegetables and produce extremely heat-resistant ascospores and are often isolated and associated with the deterioration of various products, including jams, pasteurized fruit products (e.g., juices), and canned fruits [5,6,7,8,9,10].

*M. ruber* belongs to the *Ascomycetes* class and is an important species related to food deterioration. Its main contamination source is soil, and it is mainly related to post-harvest contamination [11,12]. Its ascospores are able to develop in environments presenting low oxygen tension, low pH, and high salt concentrations. *M. ruber* was isolated for the first time from *Arauco* cultivar green olive bulk storage brine commercialized in Brazil and imported from Argentina [13]. In table olives, fruit deterioration may result in increased pH and the development of a red mycelial mat on the surface of green table olive brines [13,14,15]. In addition, the *Monascus* genus is a potential citrinin producer, a hepatonephrotoxic compound associated with disease outbreaks in both animals and humans [16,17].

In this scenario, thermal technologies are indispensable in table olive production for mitigating fungal contamination and ensuring the safety and quality of table olive products. By subjecting olives to controlled heat treatments such as sterilization and pasteurization, thermal technologies effectively reduce microbial populations, including heat-resistant fungal spores, thereby minimizing the risk of contamination during processing and storage [18].

Therefore, collaboration between industry stakeholders, regulatory agencies, and research institutions is essential to develop and implement effective control measures and ensure consumer safety and quality following sustainable production. In this context, the aim of this study was to determine the thermal resistance of *M. ruber* ascospores in canned table olive brine without preservatives. We followed the trade Standards for Table Olives [19], which indicate how table olives can be preserved through heat processes, such as pasteurization, or without the addition of preservatives. Based on the Thermal Death Tubes (TDTs) technique, the thermal inactivation by the models was evaluated to understand an effective process to eliminate this fungus without the addition of preservatives.

## 2. Material and Methods

### 2.1. Fungus and Ascospore Suspension Preparation

An ascospore suspension of *M. ruber* (IOC 4667) was prepared by growing the fungus for 30 days at 30 °C in a Roux bottle containing 200 mL of unacidified PDA (Fluka, Sigma-Aldrich, Saint Louis, MO, USA). The ascospores were harvested by adding 10 mL of sterile distilled water with 0.1% Tween 80 and scraping the PDA surface with a sterile glass rod. The suspension was filtered through sterile glass cotton to remove hyphal fragments, then homogenized using a vortex for 3 min to release the ascospores. Microscopic observation confirmed that over 90% of the ascospores were free. The suspension, containing 10^6^ ascospores mL^−1^ as measured with a Neubauer counting chamber, was stored at 4 °C and used as inoculum for all experiments.

### 2.2. M. ruber Heat Resistance Determination

Thermal resistance experiments were carried out with *M. ruber* inoculated in brine (pH = 3.5, acidified with 85% lactic acid) containing 3.5% NaCl. The brine composition was based on research by Cappato et al. [13], a study which identified the highest *M. ruber* growth in a specific brine formulation among 28 different types. For heat resistance determination, sterile Thermal Death Tubes (TDTs) were filled with 1.8 mL of brine and 0.2 mL of the ascospore suspension, achieving a final concentration of 10^5^ ascospores/mL. Sealed TDT tubes were submerged in an ultra-thermostatic water bath at 70, 75, and 80 °C for predetermined times. Post-treatment, the tubes were cooled in ice water, homogenized, and opened aseptically. Serial dilutions were plated on PDA and incubated at 30 °C for 5 days to count surviving ascospores. Survival curves were constructed by plotting log10 (CFU/mL) against time.

### 2.3. Modeling of Thermal Inactivation Kinetics of M. ruber in Brine

Inactivation models were fitted by using GInaFiT version 1.6, a freeware Add-in for Microsoft^®^ Excel, 2024 [20]. Four models (log linear, log linear + shoulder, log linear + tail, log linear + shoulder + tail) were employed to estimate key parameters by fitting experimental survival data. These parameters included *N*_0_ (initial microbial cell density [CFU/mL]), shoulder phase duration, *kmax* (kinetic inactivation rate constant [1/time unit]), and *Nres* (residual microbial cell density [CFU/mL] in the tail phase). *Dvalues* were calculated from the death rate constants (*kmax*) using the formula *Dvalue* = ln(10)/*kmax*. The z-value, representing the temperature change needed for a 1 log10 reduction in the *Dvalue*, was derived from the thermal death time (TDT) curve, constructed by regressing log10 (*Dvalue*) against corresponding temperatures. The *Dvalue* indicates the time required at a specific temperature to achieve a 1 log10 reduction in the microorganism population.

### 2.4. Model Evaluations

In the evaluation of inactivation models, predicted (*Pr*) and observed (*Ob*) microbial populations were compared. The number of data points (*n*) and the number of parameters (*p*) were also considered. The regression coefficients (*R*^2^ and *R*^2^*adj*) indicate how well the model fits the data, reflecting the deviation between observed and predicted values. The root-mean-square error (*RMSE*), a function of the sum of squared errors, measures the goodness-of-fit. High *R*^2^ and *R*^2^*adj* values, along with low *RMSE*, signify an adequate model for describing survival data [21,22].

(1) $$\mathrm{RMSE}=\sqrt{\frac{\sum {\left(Pr-Ob\right)}^{2}}{n-p}}$$

## 3. Results and Discussion

The thermal model, utilizing different temperatures, can have a role in eliminating fungal contamination in canned table olives. Subjecting olives to a series of temperatures during thermal treatments is a strategic approach aimed at determining the optimal conditions for fungal pathogen elimination while maintaining the quality and integrity of the olives. This method used in our study involves exposing olives to different temperature levels (N = 3) and durations to assess their impact on fungal viability while minimizing detrimental effects on the sensory attributes and nutritional composition of the olives [23]. 

**Figure 1:**
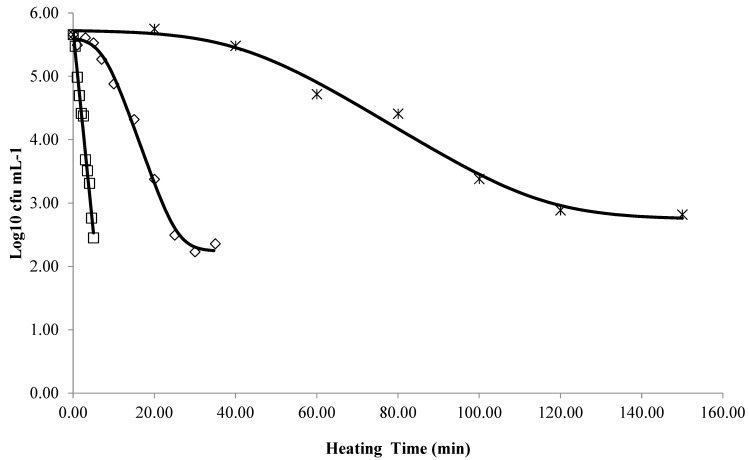
Survival curves of *Monascus ruber* ascospores in table olive brine (pH = 3.5 and 3.5% NaCl) at 70 °C (_*_), 75 °C (◊), and 80 °C (□), fitted by best models.

Table 1 presents the kinetic parameters obtained through the predictive models applied for each treatment. The model with the best fit to the experimental data was determined from statistical analysis. Figure 1 illustrates the survival curves of *M. ruber* ascospores in table olive brine (pH = 3.5 and 3.5% NaCl) at 70 °C, 75 °C, and 80 °C. In the figure, it is possible to observe that the inactivation kinetics tends to present a linear relationship with the increase in temperature.

The results (Table 1) of fitting four inactivation models to the survival data of *M. ruber* ascospores in olive brine (3.5% NaCl; pH = 3.5) at 70 and 75 °C show that the log linear + shoulder + tail model consistently provided the best fit, while for 80 °C, the best model was the log linear. These models resulted in higher *R^2^* and *R^2^adj* values and a lower *RMSE*, indicating high precision in data fitting and an effective description of survival kinetics [21,22]. The shoulder phase at 70 °C (41.5 min) and 75 °C (7.4 min) indicates a period of resistance before the inactivation rate increases. It is worth mentioning that the shoulder phase time is crucial to determine the correct binomial time and temperature for the treatment and must be added to the intensity of the heat treatment (based on Dvalue). 

The shoulder region is more pronounced at lower temperatures, while at 80 °C, the death rate follows a log-linear decrease without a tailing region, making the log linear model most appropriate. Similar survival curve behaviors were observed in other studies for *Neosartorya fischeri*, *Byssochlamys fulva*, *Byssochlamys* nivea, *Hamigera avellanea*, and *Thermoascus crustaceus* ascospores [6,8,9,24,25,26]. The final model contributes to ensuring the safety and quality of table olives by providing validated strategies for fungal control, minimizing the use of preservatives and chemical additives.

Another parameter of great relevance for determining the intensity of the heat treatment to be applied to a food is the *Dvalue* of the target microorganism. *Dvalues* for *M. ruber* inactivation in brine (Table 1) were determined from the best-fitting model for each survival curve. At 70, 75, and 80 °C, *D*-values were 24.8, 5.4, and 1.6 min, respectively. These results align with those reported by Panagou et al. [27], who found *Dvalues* of 46.08, 4.91, and 0.88 min at 70, 75, and 80 °C in brine olives (pH 3.8 and 5.6% NaCl), respectively. Differences may arise from variations in medium composition, mold strains, or methodologies used to evaluate heat resistance.

The *Nres* values, representing residual population density, were 2.75 log_10_ CFU/mL at 70 °C and 2.24 log_10_ CFU/mL at 75 °C, indicating a small but notable fraction of surviving ascospores.

These findings have significant implications for optimizing pasteurization processes in the table olive industry. The clear reduction in *D*-values with increasing temperature highlights the importance of applying higher pasteurization temperatures to ensure effective microbial control. By reducing the time required for microbial safety, higher temperatures can improve both processing efficiency and product safety. These data support the development of guidelines for thermal processing conditions aimed at achieving optimal microbial safety in canned table olives, thus contributing to the production of safer, high-quality table olives.

Knowledge of the fungal microbiota contaminating canned food and its implications is crucial for determining control strategies [5]. Owolabi et al. [1] emphasize the importance of understanding fungal contamination and mycotoxin risks in fermented foods, highlighting that heat-resistant fungi like *M. ruber* pose significant challenges in food preservation. Bouranta et al. [2] discuss quality assurance in table olive production, noting that microbial contamination control is crucial for maintaining product quality. Dijksterhuis et al. [10] provide an overview of fungal spores’ resilience, stressing the difficulty in eradicating these organisms due to their heat resistance.

Silva et al. [28] compare different methods for inactivating microbial spores, including innovative treatment, and find that heat resistance varies significantly among different species and strains. Farag et al. [29] review detection and inactivation methods for foodborne microbial spores, underscoring the need for effective preservation strategies to ensure food safety. Davies et al. [30] explore evolving challenges in fungal control within the food supply chain, recommending integrated approaches to manage fungal contamination.

*M. ruber* displays a moderate heat resistance when compared to other heat-resistant molds. Sant’ana et al. [9] reported a *δ* of 1.8 min at 95 °C for *Byssochlamys fulva* ascospores in clarified apple juice, while Ferreira et al. [31] found a high heat resistance of *B. nivea* ascospores in commercial pineapple nectar of D_104°C_ = 0.9 min and D_107°C_ = 0.6 min for *B. fulva* ascospores in commercial passion nectar.

*Neosartorya fischeri*, a mold that spoils acid foods, produces mycotoxins and displays great heat resistance. Salomão et al. [8] also reported significant heat resistance for *Neorsatorya fischeri* ascospores in various juices. In the case of pineapple juice (pH 3.4; 12 °Brix), this mold displayed D_90°C_ = 1.5 min. Houbraken et al. [32] reported extremely heat-resistant *N. fischeri* ascospores, which survived at 85 °C for 10 min. More recently, Kim et al. [6] reported that to inactivate 4.8 log of *N. fischeri*, a 90 °C treatment for 20 min was required. 

Scaramuzza et al. [26] evaluated the heat resistance of a *Hamigera avellanea* strain isolated from a spoiled semi-finished strawberry product and of a *Thermoascus crustaceus* strain from a spoiled sweetened beverage. The authors assessed the heat resistance for both molds in three heat mediums (grape juice, apple juice, and buffered glucose solution). *Thermoascus crustaceus* proved to be the most resistant in both original isolation media, presenting a D_95°C_ of 1.82 min in apple juice, while *Hamigera avellanea* presented a D_95°C_ of 0.68 min at the same temperature in the same medium. Hosoya et al. [33] reported that the numbers of spoilage incidents involving *Thermoascus* species are increasing in food industries, especially in the juice sector. 

These findings support the necessity of optimizing heat treatments to achieve effective spore inactivation while preserving product quality. By varying the temperature parameters, producers can identify the temperature threshold required to achieve effective fungal eradication without compromising product quality. Additionally, assessing the impact of different temperature treatments allows for the optimization of processing parameters to achieve maximal fungal elimination while minimizing energy consumption and processing time [34].

**Figure 2:**
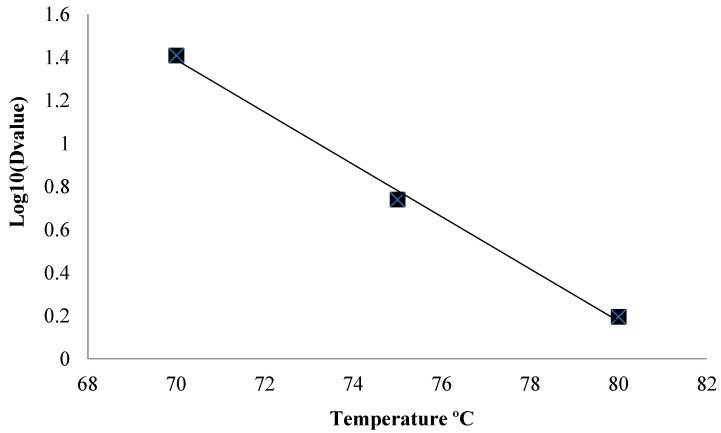
Thermal death time (TDT) curve of *Monascus ruber* ascospores in brine (pH = 3.5 and 3.5% NaCl).

Figure 2 displays the thermal death time (TDT) curves used to calculate the *z*-value. A *z*-value of 8.2 °C was estimated from the negative reciprocal of the slope and represents the temperature increase that results in a 10-fold decrease in the *D*-values. This result in comparison to the *z*-value for *M. ruber* (*z*-value = 5.8 °C) isolated by Panagou et al. [27] indicates a higher thermal resistance in the present study while using a specific *M. ruber* strain (IOC 4667). This difference may be explained by the difference in olive brine composition, in addition to strain specificities, since Panagou et al. [27] used brine containing 5.8% NaCl in comparison to the 3.5% NaCl used in the present study, indicating that the increase in % NaCl may contribute to decreased microorganism thermal resistance.

According to Sant’ana et al. [9], the efficiency of thermal processes to inactivate fungi spores is dependent on the *z*-value of the microorganisms, since small variations in temperature processes can minimally affect the heat inactivation of fungus spores, which have a low *z*-value. Thus, it is very important to control the thermal process temperature in order to prevent fungus survival and ensure the microbiological safety of the product.

Despite moderate heat resistance, *M. ruber* ascospores are capable of withstanding heat treatment comprising table olive pasteurization. According to the COI [35], table olive pasteurization should reach at least 62.4 °C for 15 min (F_62.4°C_ = 15 min), with *Propionibacterium* as the target microorganism. From the *z*-value and *D*-values, it is possible to estimate the *D*-values, for different temperatures, using Equation (2) [30].

(2) $$log\frac{DT}{D\mathrm{Tref}}=\frac{T\mathrm{ref}-T}{z}$$

where *DTref* and *DT* are the *D*-values at the reference temperature *Tref* and *T* (°C) of the isothermal treatment, respectively.

Table 2 indicates the difference between the minimum treatment required by the standard international legislation [35], based on the thermal resistance equivalent of the target microorganism (*Propionibacterium—*F_62.4°C_ = 15 min) at 70, 75, and 80 °C and the necessary treatment to inactivate one *M. ruber* log cycle, based on the *z***-**value and *D*-values obtained herein.

Table 2 provides a comparative analysis between the minimum thermal treatment required by current international legislation and the necessary treatment to inactivate *M. ruber* ascospores by one log cycle at 70 °C, 75 °C, and 80 °C and shows they are significantly lower than the times for the reduction of just 1 log cycle (*D*-values) for *M. ruber* (66.3 min at 70 °C, 12.85 min at 75 °C, and 1.6 min at 80 °C). It is worth mentioning that due to the presence of the shoulder phase at temperatures of 70 and 75 °C, the treatment to reduce 1 log cycle must be increased by the time of the shoulder phase. Therefore, it is important to know this fact, to avoid undersizing heat treatment, affecting the microbiological safety of the product.

This discrepancy highlights the need for revised thermal processing guidelines specific to *M. ruber* to ensure adequate inactivation and enhance the microbial safety of olive products. The current standards, targeting *Propionibacterium* with a *z*-value of 5.25, may not be sufficient for the more heat-resistant *M. ruber*, underscoring the importance of tailored thermal treatments in food safety protocols. Thus, the thermal process of table olives is ineffective to eliminate *M. ruber*, and its survival can result in relevant deterioration effects, such as fruit softening, the development of a red mycelial mat on the brine surface of canned table green olives, and pH increases, thus resulting in pathogen growth risks [13].

Based on our results, the importance of this approach lies in its ability to provide tailored new solutions for fungal control, considering the specific heat resistance of *M. ruber* in olive products without preservatives. Additionally, utilizing a range of temperatures and times higher than those indicated by regulatory agencies can offer new insights into the kinetics of thermal inactivation, helping producers optimizing processing parameters to achieve maximum efficacy in fungal elimination. Similar results and conclusions were reported by Kim et al. [6] for *N. fischeri* inactivation. Those authors indicated that the commercial pasteurization of apple juices (77–88 °C for 25–30 s) was far from the minimum required to avoid *N. fischeri* contamination.

Tranquillini et al. [36] reported that, due to high thermal resistance, traditional pasteurization processes would be insufficient to inactivate *Thermoascus* species, such as *T. trachyspermus*, leading to potential spoilage problems. In this context, some spoilage incidents involving *Thermoascus* species have been observed in the food industry [33]. Consequently, knowledge of mold heat resistance in acidic foods is important, as it allows for reassessments concerning the effectiveness of traditional pasteurizing processes and aids in the development of effective contamination avoidance processes.

Carefully selecting the temperature regimes over time allows producers to ensure that the thermal treatment effectively eliminates fungal pathogens while preserving the natural attributes of the olives. This approach helps maintain consumer acceptance and satisfaction without the need for antifungal chemical agents. This is particularly crucial in the current market where there is a growing consumer demand for natural and clean-label products, free from synthetic additives. Our formulation, devoid of preservatives, exemplifies this trend and underscores the necessity of optimizing pasteurization methods to ensure both safety and quality.

Additionally, optimizing pasteurization methods addresses microbial safety concerns and enhances the overall sustainability of the production process. Fine-tuning the temperature and duration of pasteurization can significantly reduce energy consumption, thereby lowering the environmental impact of production. This improvement not only aligns with sustainable food production goals but also contributes to the efficiency and cost-effectiveness of the operation. Sustainable practices in food processing are increasingly important as they support environmental stewardship while ensuring the economic viability of food production systems.

Studies by Owolabi et al. [1] and Bouranta et al. [2] reinforce the need for stringent microbial control measures in food products, especially those free from chemical preservatives. They highlight the risks associated with fungal contamination and the subsequent production of mycotoxins, which can pose significant health risks. Dijksterhuis et al. [10] also emphasize the resilience of fungal spores and the challenges they present in food preservation.

## 4. Conclusions

Different temperature settings during thermal treatments for olives are a smart strategy to tackle *M. ruber* while preserving olive quality. Our findings show that current processes and legislation do not sufficiently eliminate this heat-resistant mold, which can cause fruit softening, pH increases, and red mycelial mats, compromising safety and quality. 

This highlights the urgent need for improved processing protocols in the Brazilian and global olive industries without relying on chemical preservatives. Detailed knowledge of *M. ruber*’s behavior is crucial for refining procedures and updating regulations. 

For future studies, non-thermal preservation techniques should be explored, such as high-pressure processing, UV treatment, and natural antimicrobial agents, to complement thermal treatments without compromising product quality.

## Figures and Tables

**Table 1:** Model parameters obtained by fitting four models to survival data of *Monascus ruber* ascospores on table olive brine (3.5% NaCl; pH = 3.5) at 70, 75, and 80 °C.

		Statistic Indexes			Kinetic Parameter Values				
Temperature (°C)	Model Type	*R* ^2^	*R* ^2^ *_adj_*	*RMSE*	*k* (min^−1^)	*D_value_* (min)	*Shoulder* *Phase* (min)	(*N_res_*)	(*N*_0_)
70	Log linear	0.935	0.924	0.338	0.05(0.00)	43.15(1.36)	-	-	6.04(0.01)
	Log linear + shoulder	0.950	0.930	0.323	0.06(0.00)	37.46(1.28)	23.0(2.32)	-	5.81(0.10)
	Log linear + tail	0.940	0.916	0.355	0.06(0.00)	39.95(2.33)	-	2.44(0.27)	6.11(0.06)
	**Log linear + shoulder + tail**	**0.989**	**0.981**	**0.171**	**0.09(0.01)**	**24.81(1.20)**	**41.5(2.34)**	**2.75(0.05)**	**5.72(0.06)**
75	Log linear	0.957	0.952	0.309	0.26(0.02)	8.82(0.77)	-	-	5.85(0.15)
	Log linear + shoulder	0.961	0.951	0.313	0.28(0.01)	8.23(0.44)	2.8(1.20)	-	5.71(0.19)
	Log linear + tail	0.970	0.963	0.272	0.30(0.04)	7.79(1.08)	-	2.09(0.03)	5.95(0.20)
	**Log linear + shoulder + tail**	**0.996**	**0.994**	**0.112**	**0.42(0.02)**	**5.45(0.30)**	**7.4(1.49)**	**2.24(0.07)**	**5.60(0.20)**
80	**Log linear**	**0.988**	**0.987**	**0.123**	**1.47(0.18)**	**1.57(0.21)**	**-**	**-**	**5.71(0.32)**

Results are average values of two independent experiments (mean of two replications + standard error). Statistic indexes: *R*^2^*:* regression coefficient; *RMSE:* root-mean-square error; *R*^2^*_adj_*: adjusted coefficient. Kinetic parameter values: ***k_max_***: kinetic inactivation rate constant (min^−1^); ***D_value_***: decimal reduction time (min); ***Nres*** and ***N*_0_**: residual and initial population density (log_10_ CFU.mL^−1^), respectively.

**Table 2:** A comparison between the minimum thermal treatment required according to the standard international legislation at 70, 75, and 80 °C and the necessary treatment to inactivate 1 log cycle of *Monascus ruber* ascospores.

Microorganism	Temperature (°C)	Minimum Treatment Required by Current Legislation * (min)	Necessary Time (min) for Reduction of 1 log Cycle
*Monascus ruber*	70	1.8	66.3
	75	0.4	12.85
	80	0.1	1.6

* Target microorganism—*Propionibacterium*—*z*-value _=_ 5.25.

## Data Availability

The original contributions presented in the study are included in the article, further inquiries can be directed to the corresponding authors.
